# CircHLA-C: A significantly upregulated circRNA co-existing in oral leukoplakia and oral lichen planus

**DOI:** 10.1080/15476278.2023.2234504

**Published:** 2023-08-02

**Authors:** Jingwen Yang, Yuhan Song, Siming Xu, Shuyun Ge, Zhou Haiwen

**Affiliations:** a Department of Oral Mucosal Diseases, Shanghai Ninth People’s Hospital, Shanghai Jiao Tong University School of Medicine, Shanghai, China; b College of Stomatology, Shanghai Jiao Tong UniversityNational Center for Stomatology, Shanghai, China; c National Clinical Research Center for Oral Diseases, Shanghai, China; d Shanghai Key Laboratory of Stomatology & Shanghai Research Institute of Stomatology, Shanghai, China

**Keywords:** Biomarkers, CircHLA-C, circRNA, high-throughput sequencing, malignant transformation, Oral leukoplakia (OLK), Oral lichen planus (OLP), oral potentially malignant disorders

## Abstract

**Background:**

Oral leukoplakia (OLK) and oral lichen planus (OLP) are common precancerous lesions of the oral mucosa. The role of circular RNAs (circRNAs) in OLK and OLP is unclear. The aim of this study was to evaluate the circRNA expression profiles of OLK and OLP, and further explore the potential role of circRNAs in the pathogenesis of these two diseases.

**Methods:**

High throughput sequencing technology was performed to detect the differentially expressed circRNA in OLK (*n* = 6), OLP (*n* = 6), oral squamous cell carcinoma (*n* = 6), and normal oral mucosa tissues (*n* = 6). Expression of selected circRNAs was validated by qRT-PCR, enzyme tolerance assay, and Sanger sequencing. Expanded sample size validation was done in 20 tissue pairs. The biological processes and signal pathways involved in differential circRNA were analyzed by GO and KEGG enrichment. TargetScan and MiRanda were used to predict miRNAs downstream of circRNA and draw competitive endogenous RNA network diagram.

**Results:**

Forty-nine circRNAs were significantly altered in OLK and OLP, including 30 upregulated and 19 downregulated circRNAs. The five selected circRNAs were validated by qRT-PCR, Sanger sequencing, and RNase R assay. GO and KEGG analyses indicated that the upregulated circHLA-C may be involved in the biological process of immune function of OLK and OLP. Bioinformatics analysis indicated that circHLA-C may be involved in the progression of OLK and OLP as a ceRNA. In validation with expanded sample size, PCR results showed that circHLA-C expression was significantly upregulated in OLK and OLP. ROC analysis indicated that circHLA-C has potential diagnostic value with good accuracy and specificity.

**Conclusion:**

Our study revealed that circHLA-C is the most significantly upregulated circRNA co-existing in OLK and OLP, and we preliminarily discuss the role of circHLA-C in the etiopathogenesis and progression of OLK and OLP.

## Introduction

Oral potentially malignant disorders is a term that refers to a morphological change in the oral mucosal tissue, which is more likely to transform into oral cancer than normal oral mucosa.^1,2^ The most important of these changes is oral leukoplakia (OLK), which is clinically characterized by a white patch or plaque that occurs on the oral mucosa that cannot be erased, and cannot be diagnosed clinically or pathologically as any other disease. Epidemiology shows that the incidence of malignant transformation is as high as 1.1%–40.8%.^3^ Oral lichen planus (OLP) is a chronic inflammatory oral mucosal disease, which is the most common potentially malignant oral mucosal disease, with a malignant transformation rate of 1.14%.^4^ The pathogenesis of OLK and OLP is complex and unclear. It is important to study the pathogenesis of OLK and OLP and identify potential molecular biomarkers for early identification at the stage of potentially malignant disease of the oral cavity to reduce the occurrence of oral cancer.

Circular RNAs (circRNAs) are a class of single-stranded RNA molecules with covalently closed loops, produced by reverse splicing of mRNA precursors (pre-mRNAs). They are abundant in eukaryotes, evolutionarily conserved, and are involved in mediating the onset and development of multiple diseases through regulation of gene transcription, translation, and splicing steps.^5^ Based on the genetic origin of circRNA sequences, circRNAs can be classified into: exonic circRNA, intronic circRNA, antisense circRNA, and sense overlapping circRNA.^6–8^ CircRNAs are highly stable and naturally resistant to exonuclease R (RNase R) due to the absence of 5’cap structure or 3’ adenylate tail, which can accumulate at high levels and may change as the disease progresses.^9,10^ CircRNAs are ideal candidates as future diagnostic biomarkers and therapeutic interventions. Of note is the presence of a large number of miRNA binding sites on circRNAs, which can act as competing endogenous RNA (ceRNA). CircRNAs can trap and bind miRNA through the “sponge” mechanism, which in turn relieves the repressive effect of the miRNA on its target gene and increases the expression level of the target gene.^11^

In recent years, a large number of studies have found that circRNA is abnormally expressed in cancer, such as gastric cancer, colorectal cancer, breast cancer, lung cancer.^12–15^ Studies have shown that circRNA is as an effective diagnostic biomarker and therapeutic target in oral submucous fibrosis and oral squamous cell carcinoma (OSCC).^16,17^ At present, there is no reliable clinical biomarker for early diagnosis and treatment of OLK and OLP. The aim of this study was to define the expression profile of circRNA in OLK, OLP, OSCC, and normal oral mucosa (NOM) tissues by high-throughput sequencing, verify and analyze the significantly differently expressed circRNAs, and preliminarily explore the co-expression and underlying mechanism by which circRNA mediates the occurrence and development of OLK and OLP.

## Results

### Expression profiles and characteristics of differentially expressed circRNAs

**Figure 1.**
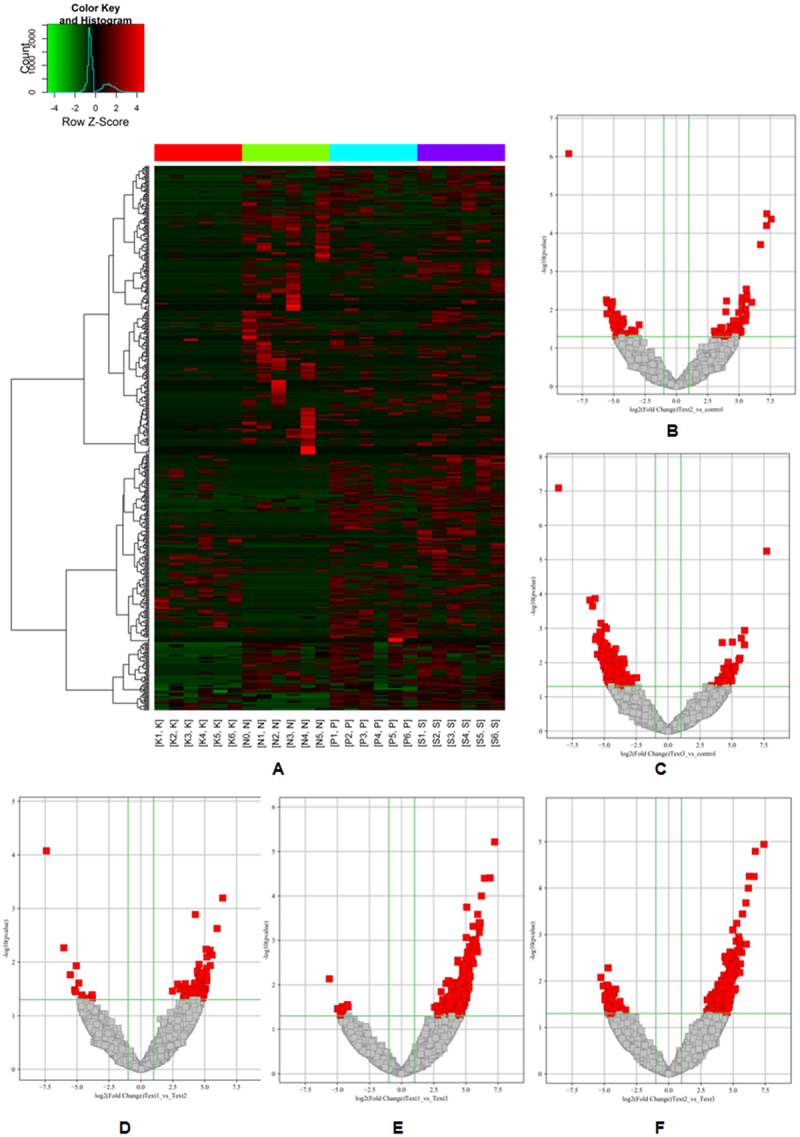
Expression profiles of circRnas.

This experiment compared the expression of circRNA in OLK (K), OLP (P), OSCC (S), and NOM (N). Hierarchical cluster analysis heat map and volcano map were painted with statistical criteria that had been determined through FC and *P* value (Figure 1a–e). In total, 135 significantly dysregulated circRNAs were identified in the PN group, of which 83 were markedly upregulated and 52 downregulated; 366 significantly dysregulated circRNAs were identified in the KN group, of which 65 were markedly upregulated and 301 downregulated; 94 significantly dysregulated circRNAs were identified in the SP group, of which 81 were markedly upregulated and 13 downregulated; 389 significantly dysregulated circRNAs were identified in the SK group, of which 376 were markedly upregulated and 13 downregulated; and 329 significantly dysregulated circRNAs were identified in the PK group, of which 275 were markedly upregulated and 54 downregulated.

(a) hierarchical clustering analysis showed circRNA expression profiles; red represents upregulated circRNAs, and green represents downregulated circRNAs，FC ≥ 2,p < 0.05; K: OLK; N: NOM; P: OLP; S: OSCC: (b) scatter plot of differences in circRNA expression between OLP and NOM tissues; (c) scatter plot of differences in circRNA expression between OLK and NOM tissues; (d) scatter plot of differences in circRNA expression between OLP and OSCC tissues; (e) scatter plot of differences in circRNA expression between OLK and OSCC tissues; (f) scatter plot of differences in circRNA expression between OLP and OLK tissues; Red, eligible circRNAs (logFC ≥ 2.0, *P* value < 0.05);Text:OSCC;Text2:OLP;Text3:OLK.

**Figure 2.**
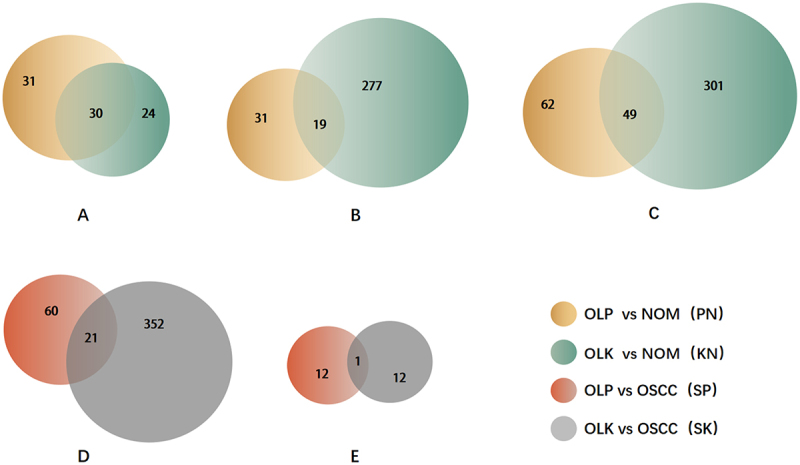
Venn diagrams of differentially expressed circRnas.

A total of 49 circRNAs (approximately 36.3%) were co-expressed differentially in the PN and KN groups, which means that 30 circRNAs that were significantly upregulated in the P group were also significantly upregulated in the K group, and 19 circRNAs that were significantly downregulated in the P group were also significantly downregulated in the K group (Figure 2a–c). In addition, 21 circRNAs that were significantly upregulated in the SP group were also significantly upregulated in the SK group, and one circRNA that was significantly downregulated in the PS group was also significantly downregulated in the KS group (Figure 2d,e).

(a) Venn diagrams of upregulated circRNAs in PN and KN; (b) Venn diagrams of downregulated circRNAs in PN and KN; (c) Venn diagrams of differentially expressed circRNAs in PN and KN; (d) Venn diagrams of upregulated circRNAs in SP and SK; (e)Venn diagrams of downregulated circRNAs in SP and SK; green, OLPvsNOM(PN); blue,OLKvsNOM(KN); red,OLPvsOSCC(SP); yellow,OLKvsOSCC(SK).

**Figure 3.**
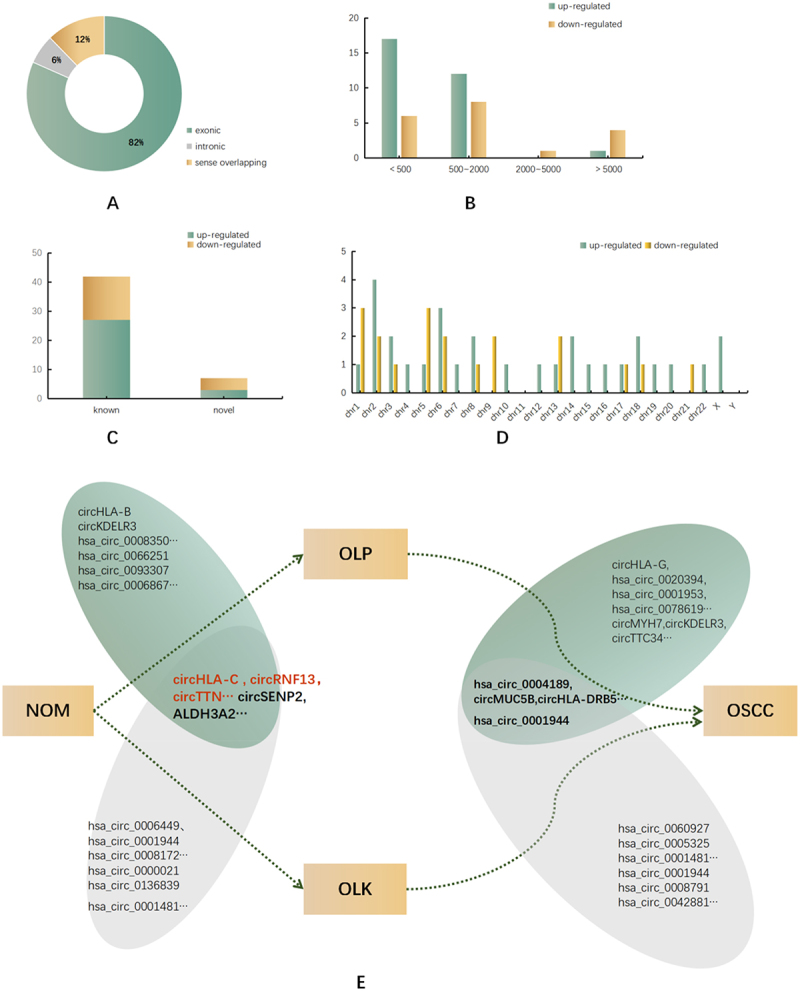
Distribution of the characteristics of significantly dysregulated circRnas.

Among the 49 circRNAs differentially expressed in the PN and KN groups, most of which were of exonic origin and had been included in the circbase, seven new circRNAs were found that had not been included in the circbase (Figure 3a, 3c). The circRNAs were concentrated in two groups: <500 and 500–2,000 nucleotides in length (Figure 3b). The circRNAs were located on almost all chromosomes, including 21 autosomes and X chromosomes (Figure 3d). Based on FC and *P* value, the circRNAs that were significantly differentially expressed in the PN and KN groups were circHLA-C, circRNF13 (hsa_circ_0006801), circTTN, circRNA circSEPN2, and circALDH3A2 (hsa_circ_0008603) as shown in Table 1.

(a) differentially expressed circRNAs in PN and KN were divided into 4 types according to the host gene structure（exon, intron and sense overlapping circRNA); (b) classification according to the circRNA length; (c) seven of the differentially expressed circRNAs were newly discovered; (d) chromosome distribution of upregulated and downregulated significantly differentially expressed circRNAs. (e) Co-expression and differential expression of circRNAs in PN and KN, SP and SK.

**Table 1.** Five circRNAs differentially expressed.

circRNA ID	Gene Name	CircBase ID	Category	Length	regulation	OLP vs NOM		OLK vs NOM		OSCC vs NOM	
						logFC	P value	logFC	P value	logFC	P vvalue
chr6:31238920–31324013-	HLA-C	novel	sense overlapping	85094	up	7.2294	0.000031	7.71865	0.000006	6.58402	0.000103
chr2:179516028–179516243-	TTN	novel	intronic	216	up	7.21315	0.000063	5.03456	0.002528	6.94575	0.000091
chr3:149613260–149639014+	RNF13	hsa_circ_0006801	exonic	379	up	5.2712	0.004797	5.7345	0.00193	5.26886	0.004744
chr17:19554860–19575269+	ALDH3A2	hsa_circ_0008603	exonic	1290	down	5.53288	0.012772	5.5323	0.005786	5.53281	0.016708
chr3:185293003–185344181+	SEPN2	novel	sense overlapping	51179	down	8.58331	0.000001	8.5832	8.12E–08	5.50212	0.016708

### Further validation of the selected circRNAs

**Figure 4.**
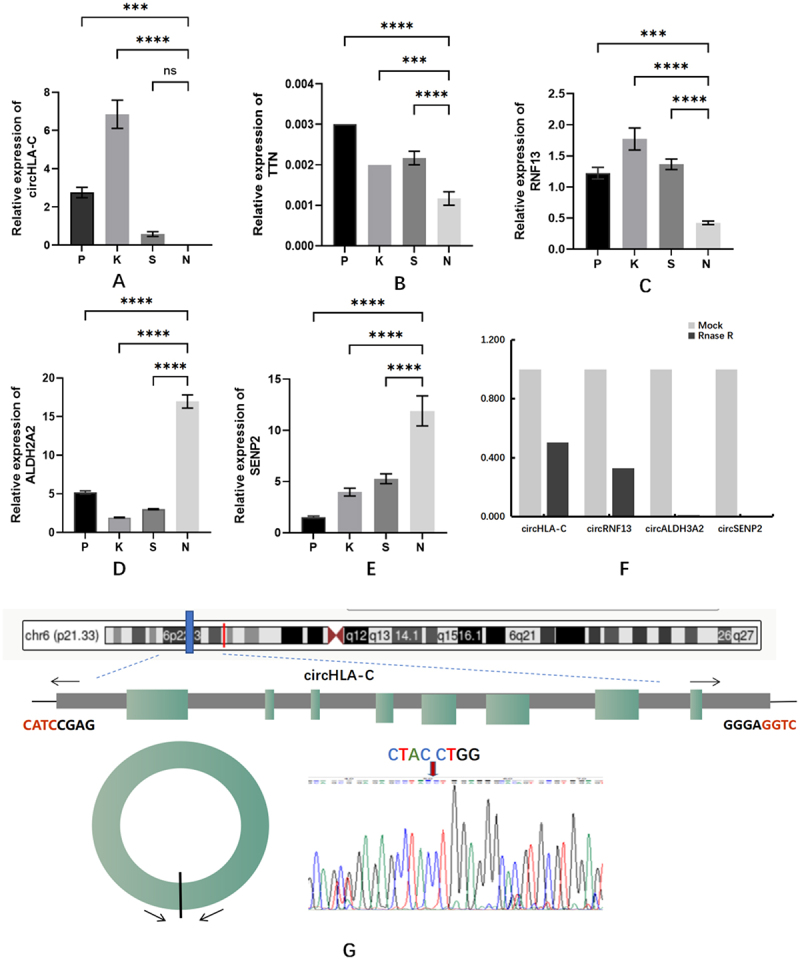
Six circRnas differentially expressed in tissues.

The above screened circRNAs were validated by qRT-PCR, and the results showed that the expression trends of the above circRNAs were the same as the sequencing results (Figure 4a–f). According to the results of the circRNAs expression, four significantly altered circRNAs were selected for enzyme tolerance assay (excluding circRNAs less than 300 nt in length), in which the FC of circHLA-C (human leukocyte antigen-C) and circRNF13 (ring finger protein 13) were 1.986 and 3.052, respectively (Figure 4g). This indicated that these two circRNAs could resist hydrolysis by RNase and were stably expressed in tissues. Sanger sequencing of circHLA-C and circRNF13 was performed, and the reverse shear site CTACCTGG was detected on circHLA-C (Figure 4h).

(a–f) five selected circRNAs were subjected to qRT⁃PCR validation in 6 OLP、6 OLK、6 OSCC and 6 NOM tissues; the five selected circRNAs (circHLA-C，circTTN，circRNF13，circALDH3A2，circSEPN2) showed the same expression trend as the sequencing results; FC ≥ 2.0，P < 0.05: ****: *P* < 0.0001; ***: *P* < 0.001; (g) RNase R analysis showed that circHLA⁃C (chr6:31238920 -31,324,013-) and circRNF13 (chr3:149613260 -149,639,014+) resisted digestion by RNase R; the relative expression of the circRNAs before and after RNase R treatment was 1.986 and 3.052 times; Back spliced site of circHLA⁃C (chr6:31238920 -31,324,013-): CTACCTGG.

### Predicting GO function and KEGG functional pathway of differentially expressed circRNAs

CircRNAs play an important role in the development of disease. GO enrichment analysis and KEGG pathway analysis were performed to predict the potential functions of circRNAs for the source genes of differentially expressed circRNAs in the PN and KN groups and SP and SK groups. GO enrichment analysis includes biological process, cellular component, and molecular function functional predictions; the most important of these is the biological process, focusing on the pathways and responses involved in the differentially expressed circRNA-derived genes.

**Figure 5.**
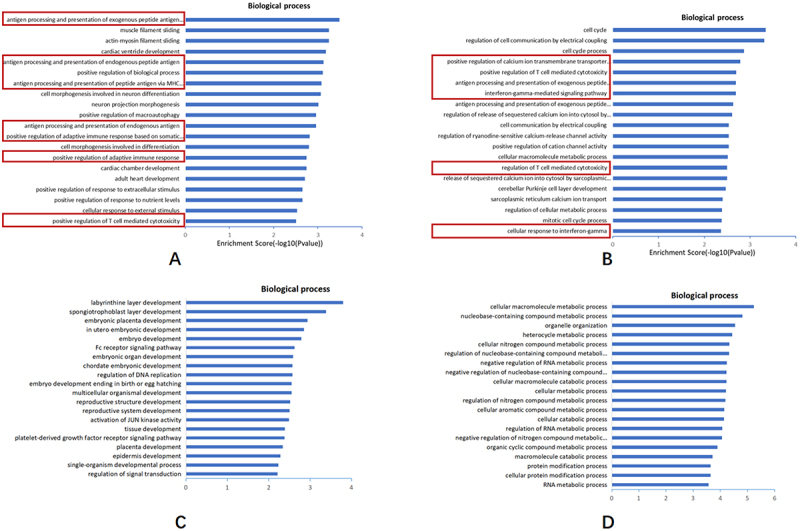
Bioinformatics analysis of significantly dysregulated expression of circRnas.

GO functional enrichment analysis of upregulated circRNA-derived genes in the PN and KN groups showed that both co-BP biological processes were enriched in 126 entries, of which upregulated pathways accounted for 114 and were associated with immune function (pathways that are commonly considered to be upregulated and enriched for activation). Notably, HLA-C was significantly enriched in these pathways. Specifically, HLA-C was involved in 6 of the top 20 upregulated BP functional entries in the KN group and 8 of the top 20 upregulated BP functional entries in the PN group, all of which were immune-related (Figure 5). The functional entries of BP shared by PN and KN groups are “antigen processing and presentation of exogenous peptide antigens via MHC class I” and “positive regulation of T cell-mediated cytotoxicity,” in which HLA-B, HLA-C, and HLA-G are jointly involved.

(a) The top 20 of biology process of upregulated circRNAs in OLP; (b) The top 20 of biology process of upregulated circRNAs in OLK; (c) The top 20 of biology process of downregulated circRNAs in OLP; (d) The top 20 of biology process of downregulated circRNAs in OLK.

**Figure 6.**
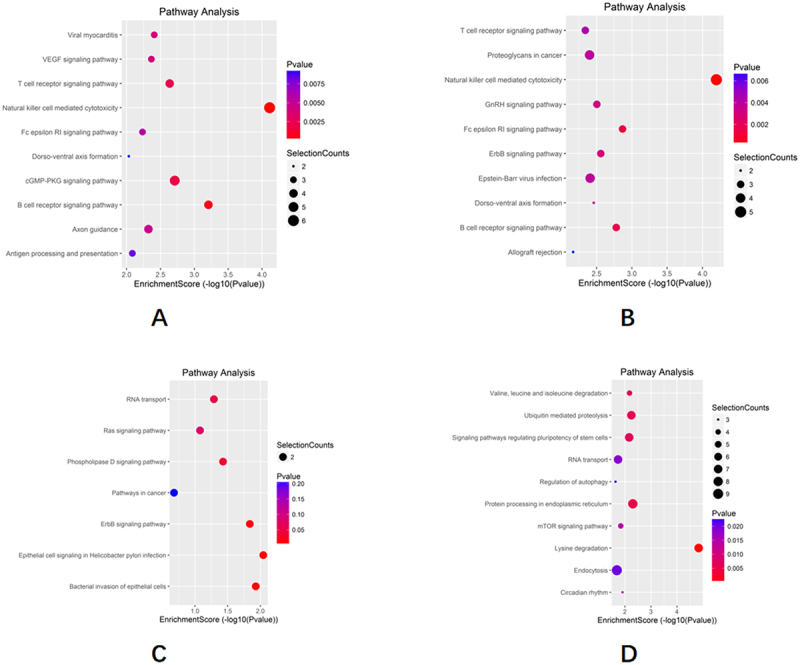
KEGG analysis of the differential expression of circRNA.

KEGG functional pathway analysis showed that a total of 82 signaling pathways were enriched in the PN group and 139 in the KN group. Of the 60 of the same pathways in the PN and KN groups, 57 were upregulated pathways (pathways that are usually considered to be upregulated and enriched for activation), and 13 were involved in HLA-C (Figure 6). Eight of the top 20 most significantly enriched pathways were involved in HLA-C, namely natural killer cell-mediated cytotoxicity, viral myocarditis, antigen processing and presentation, HTLV-I infection, allograft rejection, graft-versus-host disease, type I diabetes, and autoimmune thyroid disease. In addition, the enrichment pathways “microRNAs in cancer,” “proteoglycans in cancer,” and “pathways in cancer” were all cancer-related (Table 2).

(a) top 10 in the KEGG analysis of upregulated circRNAs in OLP; (b) top 10 in the KEGG analysis of upregulated circRNAs in OLK; (c) all of the KEGG analysis of downregulated circRNAs in OLP; (d) top 10 in the KEGG analysis of downregulated circRNAs in OLK.

**Table 2.** circRnas differentially expressed in OLP, olk and OSCC participate in KEGG.

PathwayID	Definition	Genes
hsa04650	Natural killer cell mediated cytotoxicity	HLA-B//HLA-C//MAPK1//NFATC2//PPP3CC//SOS2
hsa05416	Viral myocarditis	HLA-B//HLA-C//MYH7
hsa04612	Antigen processing and presentation	HLA-B//HLA-C//IFI30
hsa05166	HTLV-I infection	HLA-B//HLA-C//MAP2K4//NFATC2//PPP3CC
hsa05330	Allograft rejection	HLA-B//HLA-C
hsa05332	Graft-versus-host disease	HLA-B//HLA-C
hsa04940	Type I diabetes mellitus	HLA-B//HLA-C
hsa05320	Autoimmune thyroid disease	HLA-B//HLA-C
hsa04010	MAPK signaling pathway	MAP2K4//MAPK1//PPP3CC//SOS2
hsa05206	MicroRNAs in cancer	CYP24A1//FOXP1//MAPK1//SOS2
hsa05169	Epstein-Barr virus infection	HLA-B//HLA-C//MAP2K4
hsa05205	Proteoglycans in cancer	ANK1//MAPK1//SOS2
hsa05203	Viral carcinogenesis	HLA-B//HLA-C//MAPK1
hsa04514	Cell adhesion molecules (CAMs)	HLA-B//HLA-C
hsa04145	Phagosome	HLA-B//HLA-C
hsa04141	Protein processing in endoplasmic reticulum	AMFR//PDIA4
hsa05164	Influenza A	MAP2K4//MAPK1
hsa05168	Herpes simplex infection	HLA-B//HLA-C
hsa04015	Rap1 signaling pathway	LPAR3//MAPK1
hsa05200	Pathways in cancer	LPAR3//MAPK1//SOS2
hsa04144	Endocytosis	HLA-B//HLA-C

**Figure 7.**
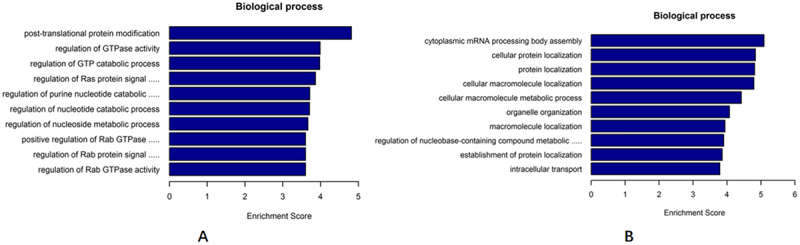
Bioinformatics analysis of significantly dysregulated expression of circRnas.

The GO functional enrichment analysis of the SP and SK groups showed 74 identical biological processes, of which 71 were upregulated pathways, including post-translational protein modification, regulation of Ras protein signaling, regulation of Ras GTPase activity, regulation of small GTPase-mediated signaling, positive regulation of Ras GTPase activity, cellular macromolecular metabolic processes, organelle organization, regulation of metabolic processes containing nucleotide-containing compounds, protein transport, regulation of nitrogen compounds metabolic processes, etc. (Figure 7). The results of KEGG functional pathway analysis showed that a total of 21 signaling pathways were enriched in the SP group, of which 20 upregulated pathways were the same pathways as in the SK group and significantly correlated with immune functional pathways. Notably, HLA-C was significantly enriched in the pathways and these pathways were similar to the above mentioned PN and KN groups intersecting KEGG pathways, including herpes simplex infection, cell adhesion molecules (CAMs), phagosomes, Epstein-Barr virus infection, viral oncogenesis, and endocytosis, in addition to the eight pathways mentioned above (Table 3).

(a) The top 10 of biology process of upregulated circRNAs in SP; (b) The top 10 of biology process of upregulated circRNAs in SK.

**Table 3.** circRNAs differentially expressed in SP and SK participate in KEGG.

PathwayID	Definition	Genes
hsa05166	*HTLV-I infection*	*AKT3//ATM//CANX//CREBBP//HLA-B//HLA-C*
hsa05169	*Epstein-Barr virus infection*	*AKT3//CREBBP//HLA-B//HLA-C*
hsa04612	*Antigen processing and presentation*	*CANX//HLA-B//HLA-C//HLA-DQB1*
hsa05416	*Viral myocarditis*	*HLA-B//HLA-C//HLA-DQB1//HLA-DRB5//ITGAL*
hsa05330	*Allograft rejection*	*HLA-B//HLA-C//HLA-DQB1//HLA-DRB5*
hsa05332	*Graft-versus-host disease*	*HLA-B//HLA-C//HLA-DQB1//HLA-DRB5*
hsa04145	*Phagosome*	*CANX//DYNC1H1//DYNC1LI1//HLA-B//HLA-C*
hsa04144	*Endocytosis*	*AP2A2//CBLB//DNAJC6//EPS15//FGFR2//HLA-B//HLA-C*
hsa04940	*Type I diabetes mellitus*	*HLA-B//HLA-C//HLA-DQB1//HLA-DRB5*
hsa03013	RNA transport	CLNS1A//KPNB1//NUP98//PABPC1//RGPD1
hsa05320	*Autoimmune thyroid disease*	*HLA-B//HLA-C//HLA-DQB1//HLA-DRB5*
hsa04514	*Cell adhesion molecules (CAMs)*	*HLA-B//HLA-C//HLA-DQB1//HLA-DRB5*
hsa04810	Regulation of actin cytoskeleton	ARHGEF7//FGFR2//ITGAL//ITGB6//PIK3CA
hsa05164	Influenza A	AKT3//CREBBP//HLA-DQB1//HLA-DRB5//NUP98//PIK3CA
hsa05168	*Herpes simplex infection*	*CREBBP//HLA-B//HLA-C//HLA-DQB1*
hsa05203	*Viral carcinogenesis*	*CREBBP//HLA-B//HLA-C//PIK3CA*
hsa05202	Transcriptional misregulation in cancer	ATM//ETV6//PTK2//WHSC1//ZEB1
hsa04650	*Natural killer cell mediated cytotoxicity*	*HLA-B//HLA-C//ITGAL//PIK3CA*
hsa04141	Protein processing in endoplasmic reticulum	CANX//SEC31A//UBQLN1//UGGT2
hsa04510	Focal adhesion	AKT3//ITGB6//PIK3CA//PTK2

### ROC curve analysis of circHLA-C in OLK and OLP

**Figure 8.**
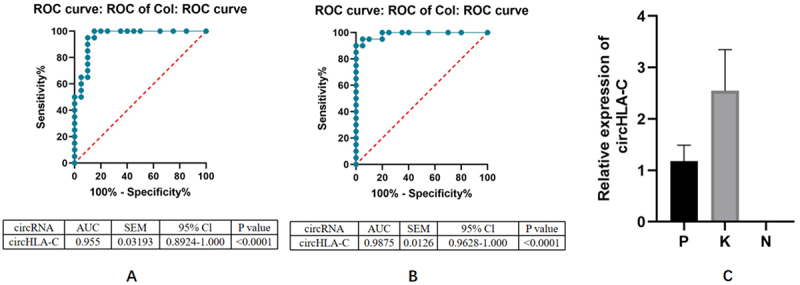
ROC curve analysis of circHLA-C in OLK and OLP.

Expanded sample size validation by qRT-PCR for circHLA-C in OLK (n = 20) and OLP (n = 20), with NOM (n = 20) as the control group was performed. The ROC curve analysis was performed to evaluate the potential diagnostic value of circHLA-C. The area under curve (AUC) of circHLA-C in the KN group was 0.9550 (95% confidence interval [CI]: 0.894–1.000, P < 0.001), and the AUC of circHLA-C in the PN group was 0.9875 (95% CI, 0.9628–1.000, *P* value < 0.0001). In general, the larger the AUC, the stronger the diagnostic power, suggesting that circHLA-C has the potential to become a biomarker for OLK and OLP (Figure 8).

(a) The ROC curves showed that the expression of circHLA-C has potential value in the diagnosis of OLK. (b) The ROC curves showed that the expression of circHLA-C has potential value in the diagnosis of OLP; (c) circHLA-C were subjected to qRT⁃PCR validation in 20 OLP and 20 OLK. ROC, receiver operating characteristic; AUC, area under curve; SEM, standard error of mean; CI, confidence interval.

### CircRNA/miRNA interaction analysis

**Figure 9.**
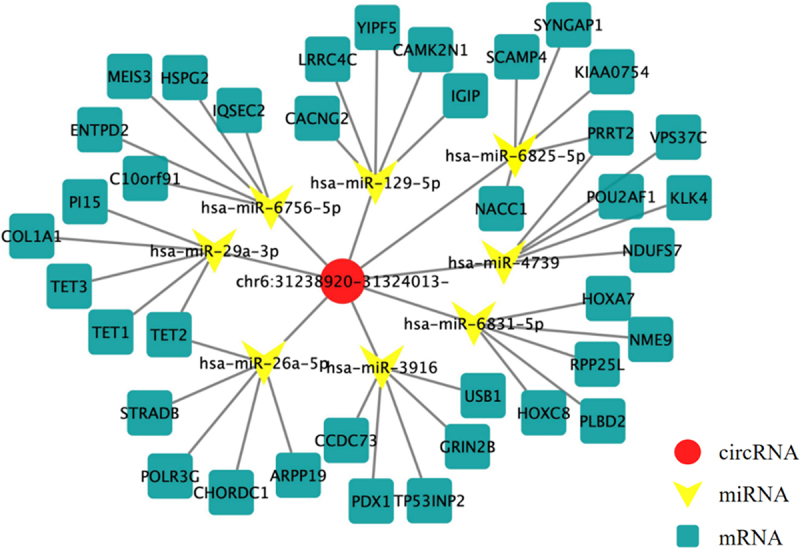
CeRNA interaction. ceRNA: competing endogenous RNA of circHLA-C; Red:circHLA-C; Yellow:microRNA; Blue:mRNA.

circRNAs can act as competitive endogenous RNAs to regulate gene expression by adsorbing miRNAs through a “sponge mechanism.”^10^ Possible interactions between circHLA-C and miRNAs were predicted by TargetScan and miRanda analysis. Using cytoscape software, a ceRNA network diagram that can show the interaction relationship was constructed based on published mRNAs and miRNAs associated with carcinogenesis (Figure 9). CircHLA-C can interact with hsa-miR-4739, hsa-miR-3916, hsa-miR-6756-5p hsa- miR-6825-5p, hsa-miR-6831-5p, hsa-miR-26a-5p, hsa-miR-129-5p, and hsa-miR-29a-3p, acting as a “sponge.”

## Discussion

OLK and OLP are common mottled diseases of the oral mucosa, with white damage being the main clinical manifestation. Moreover, both these entities are at risk of malignant transformation, i.e., they are important potentially malignant diseases of the oral mucosa.

Commonalities and differences between the two diseases, as well as an evolution from one disease to the other have been found. In some cases, the pathological diagnosis is “oral leukoplakia,” even though it is a typical reticular OLP; in some cases, the pathological diagnosis is “oral lichen planus,” even though it is a typical plaque OLP. In addition, it has been found that the two diseases sometimes coexist in the same patient with the presence of both diseases in the oral cavity, or the appearance of both diseases at different times. Many scholars have also noted an interesting phenomenon, with partial damage appearing as an evolution from OLP to OLK; for example, Garcia-Pola monitored the evolution of 14 (2.7%) patients with OLP to proliferative verrucous oral leukoplakia in 515 patients followed for a mean of 14.5 years;^18^ Chainani-Wu N summarized 9 cases diagnosed with lichen planus, after the first clinical follow-up followed by localized leukoplakia at intervals of 1.5 to 6.5 years.^19^ Pathological diagnostic issues have also been reported in the literature with OLP and OLK sometimes being difficult to distinguish. Gao Yan et al.^20^found OLK-like pathological changes in the process of OLP carcinoma: among 19 cases of OLP, 9 cases were found to be accompanied by leukoplakia-like pathological manifestations. Among them, one case was diagnosed as OLP at the initial biopsy, leukoplakia with abnormal epithelial hyperplasia at the second biopsy, and confirmed as carcinoma at the third biopsy. Interestingly, lesions in the tissues surrounding the cancerous area were found to manifest as leukoplakia in 19 cases of OLP cancerous tissue. This has prompted us to speculate whether these two diseases are in fact intrinsically linked. Are there similar or different pathways in the development and carcinogenesis potential of these two diseases? What are the commonalities and differences in the expression and role of circRNAs in these two diseases?

CircRNAs are involved in genesis of various disease and oral cancer progression through transcriptional and post-transcriptional processing modifications.^21,22^ There is a paucity of literature on the regulatory mechanisms of circRNAs in the development of OLP and OLK. Previous research results of our group showed that circRNAs are significantly differentially expressed in OLP and OLK tissues, and in the course of further in-depth studies, we found that the processes of these two diseases may have certain commonalities.

1. There was partial overlap between OLK and OLP in differentially expressed circRNAs related to their development and carcinogenesis potential: some circRNAs were significantly differentially expressed in both OLP and OLK. About 36.3% of circRNAs were the same in the PN and KN groups. Thirty circRNAs were upregulated in both the PN and KN groups, and 19 circRNAs were downregulated in both the PN and KN groups, such as circHLA-C, circTTN, circRNF13, circUGGT2. About 23.2% of the differentially expressed circRNAs in the SP group were the same as those in the SN group, of which only one circRNA was downregulated and the remaining 21 were all upregulated circRNAs, such as hsa_circ_0004189, circMUC5B. Available literature suggests that circHLA-C plays an important role in lupus nephritis through the sponge miR-150;^23^ circTTN promotes proliferation and differentiation of myogenic cells through IGF2/PI3K/AKT signaling pathway as miR-432 sponge (a);^24^ circRNF13 inhibits proliferation and metastasis of nasopharyngeal carcinoma through SUMO2;^25^ circUGGT2 (hsa_circ_0030632) is associated with colorectal and hepatocellular carcinoma.^26,27^
2. CircHLA-C is the most differentially expressed circRNA with the highest fold change among circRNAs that are simultaneously upregulated in OLK and OLP. CircHLA-C expression was low in NOM and significantly higher in OLP and OLP tissues, and the relative expression of circHLA-C was significantly higher in OLK than in OLP and OSCC tissues, with NOM<OSCC<OLP<OLK. Expanded sample size validation and ROC curve analysis to assess the potential diagnostic power of circHLA-C in OLK and OLP were performed. The results indicate that circHLA-C has high specificity and sensitivity for the diagnosis of OLK and OLP. In addition, circHLA-C expression was positively correlated with abnormal OLK development, and the difference between circHLA-C expression and mild and moderate atypical hyperplasia of OLK was statistically significant. CircHLA-C expression, hence, plays an important role in the progression of OLK to OSCC.^28^
3. Bioconductivity analysis revealed that there may be many common functional pathways in the development of OLP and OLK, which are significantly associated with immune function, and that the common BP functional entry “positive regulation of T-cell-mediated cytotoxicity” is shared by both HLA-B and HLA-C. The human leucocyte antigen (HLA) is the major human histocompatibility complex (MHC), controlled and produced by the HLA gene, which is present on the surface of cells and regulates the specific immune response of the body, and is the basis by which the immune system differentiates between self and foreign substances, and these molecules can be subdivided into HLA-A, HLA-B, and HLA-C.^29,30^ Recent studies have found that specific expression of HLA-C-restricted T-cell receptors (TCRs) in vivo by infusion of autologous T cells can be targeted for the treatment of pancreatic cancer.^31^ Inflammatory mediators and infiltrating immune cells were also found to play an important role in the progression of OLC and OLP, with CD4+ T lymphocytes, Foxp3+ regulatory T cells, CD68+ macrophages, and IL-4 co-expressed in OLP and OLK,^32,33^ presumably regulating the expression of genes specific to immunity; this is one of the main reasons for the development of OLP and OLK.
4. Many common functional pathways may also exist in the development and carcinogenesis potential of OLP and OLK, and GO functional enrichment analysis of the SP and SK groups showed that the development of both diseases was significantly associated with Ras signaling, cell differentiation proliferation, and apoptosis. Analysis of KEGG functional pathways showed that all 20 upregulated pathways in the SP group were shared with the SK group, and notably, HLA-C was found to be significantly enriched in these pathways as well, including herpes simplex infection, CAMs, phagosomes, Epstein-Barr virus infection, viral oncogenesis, and endocytosis, in addition to the intersecting KEGG pathways in the PN and KN groups mentioned above.
5. CircHLA-C may interact with a variety of cancer-related miRNAs, and the most studied mechanism of circRNA action in disease development is the ceRNA mechanism of circRNAs, which can act as a “sponge” for microRNAs.^34^ In this study, we found that circHLA-C can act on cellular regulatory networks by binding to miRNAs, such as hsa-miR-26a- 5p, hsa-miR-129-5p, hsa-miR-29a-3p, and hsa-miR-4739 through complementary pairing. According to the now published mRNA and miRNA literature related to carcinogenesis, circGPR137B was found to be downregulated or miR-4739 upregulated in hepatocellular carcinoma. CircGPR137B acted as a sponge for miR-4738 and upregulated its target gene, and ectopic expression of circGPR137B significantly inhibited hepatocellular carcinoma growth and metastasis^35^; in OLK and cancer tissues, the miR-26a and miR-29a expression was significantly downregulated but upregulated in OLP^36^; miR-129-5p expression level was significantly downregulated in OLK-OSCC tissues.^37^

To our knowledge this is the first study that shows that there are some circRNAs differentially expressed in OLK and OLP tissues showing similar trends. CircHLA-C is the most significantly differentially expressed circRNA in both diseases. GO functional analysis and KEGG pathway enrichment revealed that circHLA-C plays a key role in cellular immunity in OLK and OLP. In conclusion, circHLA-C may be a valuable biomarker for the diagnosis of OLK and OLP, and can potentially provide new insights for future diagnostic and prognostic studies on OLK and OLP.

## Methods

### Patients and samples

One hundred and six specimens, including OLK (*n* = 26), OLP (*n* = 26), OSCC (*n* = 26), and NOM tissues (*n* = 26), were obtained from the Department of Oral Mucosa and the Department of Oral and Maxillofacial Head and Neck Tumor Surgery of the Ninth People’s Hospital of Shanghai Jiaotong University School of Medicine from September 2018 through November 2018. The patients were diagnosed by histopathological examination based on the diagnostic criteria of the World Health Organization. All tissue specimens were frozen in liquid nitrogen or preserved in a −80°C refrigerator immediately after acquisition until RNA extraction. None of the patients had used local or systemic glucocorticoids or had severe cardiovascular system disease or any liver, kidney, or other organ dysfunction. All patients participated voluntarily in this study and signed informed consent forms prior to surgery. The study was approved by the Ethics Committee of The Ninth People’s Hospital of Shanghai [No.89(2012)21]. OLK (*n* = 6), OLP (*n* = 6), OSCC (*n* = 6), and NOM (*n* = 6) tissues were selected from the collected samples for high-throughput sequencing (see Table 4 for sample characteristics), and the sequencing results were analyzed by the following comparisons: Differentially expressed circRNAs of the OLP group and NOM group were recorded as: PN group; Differentially expressed circRNAs of the OLK group and NOM group were recorded as: KN group; Differentially expressed circRNAs of the OSCC group and OLP group were recorded as: SP group; Differentially expressed circRNAs of the OSCC group and OLK group were recorded as: SK group.

**Table 4.** Patient and control characteristics.

	Number	Age (years)	Sex	Location
NOM	N/N0	24.67 ± 5.28	F	Gingiva
	N1		F	Buccal
	N2		F	Buccal
	N3		M	Buccal
	N4		F	Buccal
	N5		M	Lip
OLP	P1	39.83 ± 12.73	F	Buccal
	P2		F	Tongue
	P3		F	Tongue
	P4		M	Tongue
	P5		M	Tongue
	P6		M	Tongue
OLK	K1	53.5 ± 10.03	F	Tongue
	K2		F	Tongue
	K3		M	Tongue
	K4		F	Tongue
	K5		M	Tongue
	K6		F	Buccal
OSCC	S1	63.83 ± 17.08	M	Floor of mouth
	S2		M	Tongue
	S3		M	Tongue
	S4		F	Tongue
	S5		M	Tongue
	S6		F	Tongue

### RNA extraction and quality control

Total RNA was extracted using TRIzol reagent (Life Technologies, Carlsbad, CA, USA). The RNA concentrations for each specimen were measured using a NanoDrop ND-1000 (Thermo Fisher Scientific, Waltham, MA, USA). Spectrophotometer OD260/OD280 values were used for assessing RNA purity indexes. Quality control results indicated a range of OD260/OD280 between 1.8 and 2.1. Sufficient RNA was used with Ribo-Zero rRNA Removal Kits (Illumina, USA) to eliminate ribosomal RNA (rRNA) according to the kit instructions. RNA libraries were constructed using rRNA-depleted RNAs and TruSeq Stranded Total RNA Library Prep Kits (Illumina). RNA libraries were adjusted to 10 pM and converted into denatured single-stranded DNA molecules and amplified *in situ* as hierarchical clusters. Sequencing libraries were detected by an Agilent 2100 Bioanalyzer using an Agilent DNA 1000 chip kit (Agilent, Technologies, USA).

### RNA library construction and circRNA sequencing analysis

After sequencing by Illumina HiSeq 4000 sequencer, double-ended reads were harvested. CloudSeq Biotech (Shanghai, China) undertook the RNA library preparation and high-throughput sequencing. Quality control was performed using Q30, de-junctioning using cutadapt software (v1.9.3) to remove low quality reads and obtain high quality reads, and high quality reads were compared to the reference genome or transcriptome using STAR software (v2.5.1b). We used DCC software (v0.4.4) for circRNA detection and identification. The identified circRNAs were annotated using the circBase database and circ2Traits. Data normalization and differentially expressed circRNA screening were performed using edgeR software (v3.16.5).

Unprocessed and analyzed sequencing data, after standardization, were uploaded to the National Center for Biotechnology Information Gene Expression Omnibus (GEO). The approved GEO accession number is GSE131567, GSE131568 and GSE131182.

### Quantitative real-time RT-PCR

The PN and KN groups were screened for co-expression of differential circRNAs according to the difference ploidy and P-value, and the five circRNAs selected from the sequencing results with a significant degree of expression difference were validated by real-time quantitative RT-PCR (the larger was the fold change [FC], the smaller the P-value, and the more significant the original signal expression value, was, the better). Total RNA was extracted from tissues using TRIzol lysate, and the extracted RNA was reverse transcribed into cDNA library using SuperScript III reverse transcription kit. GAPDH was selected as the internal reference and as the endogenous reference gene for circRNA. The cDNA and internal reference were subjected to real-time quantitative RT-PCR reaction by QuantStudio 5 Real-Time PCR System (Thermo Fisher Scientific, Chino, CA, USA). The primer sequences used in this study were provided by Shanghai Biotechnology (see Table 5 for primer sequences). The data analysis was performed using the 2^−∆∆CT^ method.

**Table 5.** The primers used for qRT⁃PCR experiments.

circRNA ID	Primer Type	Primer Sequence (5”−3”)
circHLA-C (chr6:31238920–31324013-)	Forward	CGGCAAGGATTACATCGC
	Reverse	CCTTCCCGTTCTCCAGGT
circTTN (chr2:179516028–179516243-)	Forward	CCTGCTAAAGTGCCTGAAGTT
	Reverse	AACCACCAGAGGCACCTTC
circRNF13 (chr3:149613260–149639014+)	Forward	GACATAGAGCTAGAAGAAACAGACT
	Reverse	GCCTTGTATCCTGCTCTCTG
circALDH3A2 (chr17:19554860–19575269+)	Forward	CTGTTGCTCACTTTCCTGGG
	Reverse	TGACTTCCTGACTGTACACATTG
circSEPN2 (chr3:185293003–185344181+)	Forward	ACAGCTGAATGGGAGTGATTG
	Reverse	GTGGCAGCACAGAACCTTC

### RNase R assay and sanger sequencing

According to the results of the circRNAs expression, four significantly altered circRNAs were selected for enzyme tolerance assay. RNeasy MinElute Cleaning Kit (Qiagen) was used for RNase R assay, and total RNA was extracted from experimental tissues and then incubated with RNase R (5 U/mg; Epicentre Technologies). Next, the back-spliced junctions of circHLA-C were verified by Sanger sequencing. Expanded sample size was used to verify circHLA-C expression in 20 tissues each of OLK, OLP, OSCC, and NOM. Data analysis for relative expression of circRNAs was performed by 2^−∆∆Ct^ measurement.

### Construction of ceRNA network and bioinformatics analysis

Potential target miRNAs of circRNAs were predicted by TargetScan. The bioinformatics software Cytoscape (v2.8.0) was used to construct the network of circRNAs and their downstream miRNAs and mRNAs. Gene ontology (GO) functional analysis and Kyoto Encyclopedia of Genes and Genomes (KEGG) pathway analysis were performed to predict the functions of the circRNAs. GO covers three domains: biological process (BP), cellular component (CC), and molecular function (MF). Using Fisher’s test, FC ≥ 2 and *P* < 0.05 was taken as statistically significant, indicating a significant difference in GO term and KEGG pathway enrichment in host genes.

### Statistical analysis

Statistical software SPSS 19.0 (SPSS, Chicago, IL, USA) and GraphPad Prism version 8.0 (Grapahpad, San Diego, CA, USA) were used for management and statistical analysis of the data. Statistical significance of differences between the two groups was performed by unpaired t-test. Quantitative data was expressed as mean ± standard error of mean (SEM). All the experiments were performed at least three times. Receiver operating characteristic (ROC) curve analysis was used to identify the sensitivity and specificity of circHLA-C.
